# The complete mitochondrial genome of *Tetraodon pustulatus* Murray, 1857 (Tetraodontiformes: Tetraodontidae)

**DOI:** 10.1080/23802359.2026.2732521

**Published:** 2026-11-12

**Authors:** Hong Zhang, Bo-Wen Xu, Cai-Hua Sha, Bao-Zheng Luo, Xuan Chen

**Affiliations:** Technical Center of Gongbei Customs, Zhuhai, China

**Keywords:** *Tetraodon pustulatus*, mitogenome, genome annotation, phylogenetic analysis, Tetraodontidae

## Abstract

The first complete mitochondrial genome (mitogenome) of *Tetraodon pustulatus* was sequenced, assembled, and annotated. The complete mitogenome is 16,471 bp in length and contains 37 mitochondrial genes, including 13 protein-coding genes (PCGs), two rRNA genes, and 22 tRNA genes, as well as one non-coding control region (D-loop). The overall nucleotide composition is A: 29.41%, T: 23.40%, C: 31.66%, G: 15.53%, with a GC content of 47.19%. Phylogenetic analysis revealed that *T. pustulatus* clustered within *Tetraodon* and was sister to *T. lineatus*, with 100% bootstrap support. This mitogenome provides a valuable molecular resource for species identification and phylogenetic studies of Tetraodontidae.

## 1. Introduction

*Tetraodon pustulatus* Murray, 1857, also known as the redline pufferfish or Cross River pufferfish, is a freshwater pufferfish endemic to the Cross River system in southwestern Nigeria. As a member of the family Tetraodontidae, it is part of freshwater benthic communities and has ornamental potential. Wild populations are declining due to pollution and habitat degradation, and the species is currently assessed as Endangered (EN) on the IUCN Red List (Olaosebikan 2020).

Mitogenomes are useful molecular markers for species identification and phylogenetic analyses due to their maternal inheritance and conserved structure (Silva-Pinheiro and Minczuk 2022). However, no complete mitogenome of *T. pustulatus* has been reported. Accurate identification of ornamental pufferfish is relevant to customs inspection and biodiversity conservation. The mitogenome data generated in this study may facilitate the conservation of wild populations in Africa and the identification of ornamental fish in China. Here, we report the complete mitogenome of *T. pustulatus*, thereby enriching the genomic resources available for Tetraodontidae and supporting conservation research.

## 2. Materials and methods

### 2.1. Sample collection and DNA extraction

In May 2025, a specimen of *Tetraodon pustulatus* was intercepted by customs officers at Gongbei Port, Zhuhai, China (113°33′E, 22°12′N). The specimen was immediately transferred to our laboratory for species identification, which was confirmed through a combination of morphological examination and mitochondrial DNA barcoding based on the 16S rRNA gene. The voucher specimen (accession number: TP20250501; Figure 1) was deposited in the Animal Quarantine Laboratory, Technical Center of Gongbei Customs (contact: Hong Zhang, E-mail: zhangh395@mail2.sysu.edu.cn). Total genomic DNA was extracted using the E.Z.N.A.^®^ Tissue DNA Kit (Omega, Norcross, GA) and assessed using 1% agarose gel electrophoresis and a NanoDrop 2000 spectrophotometer (OD260/OD280 = 1.8–2.0, OD260/OD230 ≥ 2.0).

**Figure 1.**
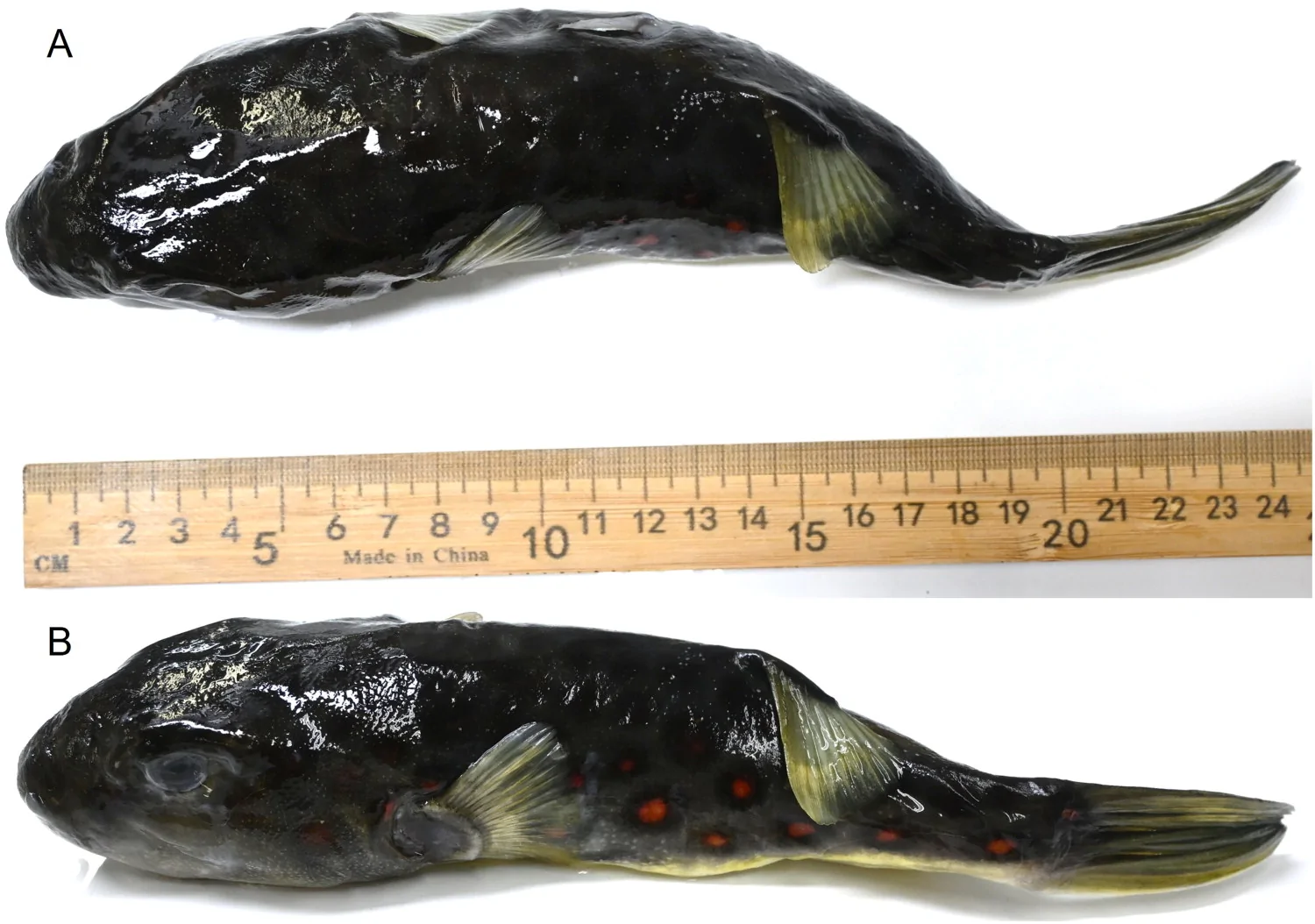
Morphological photos of voucher specimen TP20250501 of *Tetraodon pustulatus*. The specimen was intercepted during customs inspection at Gongbei Port in connection with the ornamental fish trade. Species identification was confirmed based on diagnostic red-spot morphological features and 16S rRNA barcoding. (A) Dorsal view; (B) lateral view revealing characteristic pigment markings used for taxonomic discrimination.

### 2.2. Sequencing, assembly, and annotation

DNA was fragmented to approximately 350 bp using a Covaris E220 focused-ultrasonicator, and sequencing libraries were constructed using the NEBNext Ultra™ II DNA Library Prep Kit. The libraries were sequenced on the Illumina NovaSeq 6000 platform by Sangon Biotech (Shanghai) Co., Ltd. (Shanghai, China). Raw reads were filtered with fastp v0.36 to remove low-quality reads (Q20 < 90%), adapters, and reads containing >5% ambiguous bases (N). Clean reads were *de novo* assembled using SPAdes v3.15.5 with k-mer sizes of 21, 33, 55, 77, and 99. Mitogenome contigs were identified using BLAST+ v2.15.0 against *T. lineatus* (GenBank accession no. MG913990.1). Gaps were filled using GapFiller v1.11, and sequencing errors were corrected with Pilon v1.24. Circularity was verified based on a 100 bp overlap between the two ends of the assembled sequence. Read coverage depth was calculated and visualized as a coverage depth map (Supplementary Figure S1). Genome annotation was performed using the MITOS web server, followed by manual correction through BLAST+ comparisons with published Tetraodontidae mitogenomes. A mitogenome map was generated using R (v4.0.5; Figure 2).

**Figure 2.**
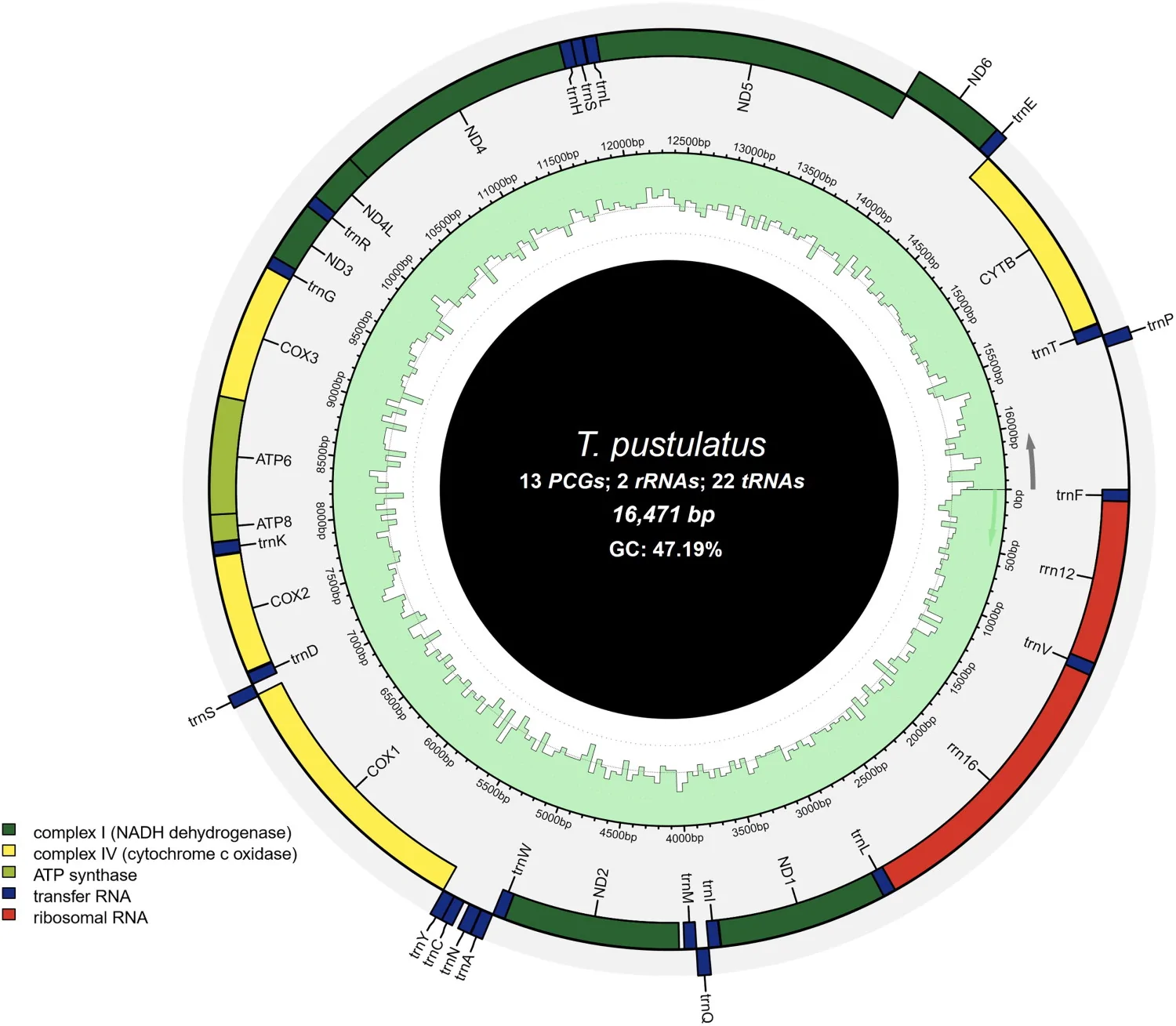
Circular mitogenome map of *Tetraodon pustulatus* (16,471 bp; GC content = 47.19%). The outer track represents genes encoded on the L-strand, and the inner track represents genes encoded on the H-strand. The pale green band indicates sequencing depth at each genomic position, generated from Illumina NovaSeq 6000 clean reads mapped to the assembled contig. Color blocks represent functional gene categories defined in the legend.

### 2.3. Phylogenetic analysis

Tetraodontiformes mitogenomes were retrieved from GenBank for phylogenetic analysis, with accession numbers and corresponding source publications provided in Supplementary Table S1 (Liu et al. 2016; Wang et al. 2016; Yamanoue et al. 2009, 2011; Yamada et al. 2021). Complete mitochondrial genome sequences were aligned using Clustal W implemented in MEGA 7. The best-fit nucleotide substitution model was selected in MEGA 7 based on the Bayesian information criterion (BIC), with GTR + G identified as the optimal model. A maximum-likelihood (ML) phylogenetic tree was constructed using the selected model with 1000 bootstrap replicates.

## 3. Results

The average sequencing coverage depth was 1891×, indicating high sequencing coverage (Figure S1). The complete mitogenome of *T. pustulatus* is 16,471 bp in length (GenBank accession no. PZ314420) and contains 37 genes (13 protein-coding genes (PCGs), two rRNAs, and 22 tRNAs), as well as one D-loop region (Figure 2). Twenty-eight genes are encoded on the heavy strand (H-strand), while the remaining nine genes (eight tRNAs and the *ND6* gene) are encoded on the light strand (L-strand). The annotated genic regions span 15,646 bp (94.99%), and non-coding regions account for 825 bp (5.01%).

Overall base composition is A: 29.41%, T: 23.40%, C: 31.66%, and G: 15.53%. The mitogenome of *T. pustulatus* exhibits a slight AT bias, with an AT content of 52.81%. Most PCGs start with ATG, except *COI* (ATC). Six PCGs (*ND2*, *ATP8*, *ATP6*, *ND4L*, *ND5*, and *CYTB*) terminate with TAA, one (*ND1*) with TAG and one (*ND6*) with AGG. The remaining five PCGs (*COI*, *COII*, *COIII*, *ND3*, and *ND4*) contain incomplete stop codons (T/TA), which are completed by polyadenylation during post-transcriptional processing (Anderson et al. 1981; Wei et al. 2022).

ML phylogenetic analysis was performed using full-length mitogenome sequences of the Tetraodontiformes taxa. The sampled Tetraodontidae species formed a monophyletic lineage that was clearly separated from the outgroup taxa belonging to Ostraciidae and Monacanthidae (Figure 3). *Tetraodon pustulatus* clustered with *Tetraodon lineatus* within the genus *Tetraodon*, with 100% bootstrap support. These results support the phylogenetic placement of *T. pustulatus*.

**Figure 3.**
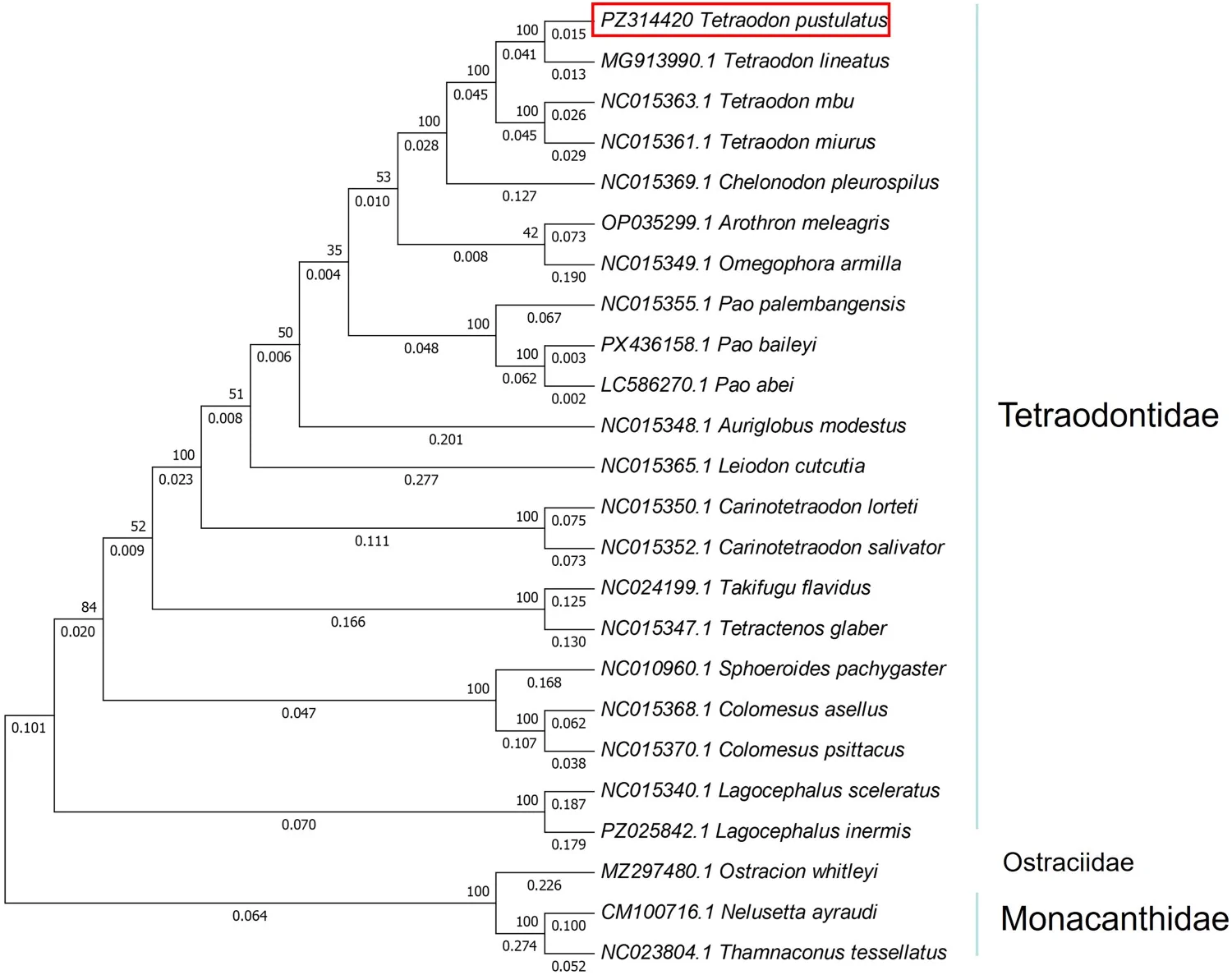
ML phylogenetic tree of Tetraodontiformes based on complete mitochondrial genome sequences, constructed in MEGA 7 using the GTR + G model and 1000 bootstrap replicates. Node numbers denote bootstrap support values, and branch lengths indicate genetic distances. The newly sequenced mitogenome (GenBank accession no. PZ314420, red box) clusters within the genus *Tetraodon*. Species from Ostraciidae and Monacanthidae were used as outgroups. All reference sequences with accession numbers and citations are listed in Supplementary Table S1.

## 4. Discussion and conclusions

The 16,471 bp mitogenome of *T. pustulatus* is comparable in size to previously reported Tetraodontidae mitogenomes (Jiang et al. 2018; Cao et al. 2020), with conserved gene arrangement reflecting stable Tetraodontidae mitogenome evolution. The slight AT bias (52.81%) is consistent with that reported for vertebrate mitogenomes and may be associated with AT nucleotide mutation rate and transcription (Formenti et al. 2021). The observed bias toward A/T-ending codons in PCGs is driven by mutation pressure (Gu et al. 2025), consistent with other Tetraodontidae. Incomplete stop codons (T/TA) identified in five PCGs are completed via post-transcriptional polyadenylation and may contribute to maintaining the compact size of the mitochondrial genome (Han et al. 2025). The phylogenetic results confirm *T. pustulatus*’ taxonomic status in *Tetraodon*, providing molecular evidence for Tetraodontidae phylogeny.

This study reports the first complete mitogenome of *T. pustulatus*. The 16,471 bp mitogenome contains 37 genes and one D-loop region, with typical Tetraodontidae characteristics. Phylogenetic analysis supports its placement within *Tetraodon*. This genome enriches Tetraodontidae genomic resources and provides valuable molecular data for species identification, phylogenetic studies, and conservation research.

## Supplementary Material

Supplemental Material

## Data Availability

The data supporting the findings of this study are openly available in GenBank of NCBI under the accession no. PZ314420. All relevant data, including datasets analyzed or generated during the study, are included in the submitted manuscript. No additional external datasets were used, and no data are withheld due to privacy or ethical restrictions.
