# Supplementary material for: The complete mitochondrial genome of Tetraodon pustulatus Murray, 1857 (Tetraodontiformes: Tetraodontidae)
Source: Mitochondrial DNA B Resour. 2026 Nov 12;11(12):1395–9. doi: 10.1080/23802359.2026.2732521 (PMC13761072; doi:10.1080/23802359.2026.2732521)
Supplement: Supplementary file 1 [file TMDN_A_2732521_SM9889.docx]

**Supplementary materials**

Table S1. GenBank Accession numbers of sequences included in this study.

| Species | Accession number | Length(bp) | References |
| --- | --- | --- | --- |
| *Nelusetta ayraudi* | CM100716.1 | 16446 | (unpublished) |
| *Takifugu flavidus* | NC024199.1 | 16449 | (Liu et al. 2016) |
| *Thamnaconus tessellatus* | NC023804.1 | 16437 | (Wang et al. 2016) |
| *Ostracion whitleyi* | MZ297480.1 | 16461 | (unpublished) |
| *Tetraodon lineatus* | MG913990.1 | 16470 | (unpublished) |
| *Tetraodon mbu* | NC015363.1 | 16508 | (Yamanoue et al. 2011) |
| *Tetraodon miurus* | NC015361.1 | 16463 | (Yamanoue et al. 2011) |
| *Arothron meleagris* | OP035299.1 | 16480 | (unpublished) |
| *Pao baileyi* | PX436158.1 | 16448 | (unpublished) |
| *Pao abei* | LC586270.1 | 16448 | (Yamada et al. 2021) |
| *Pao palembangensis* | PX436159.1 | 16481 | (unpublished) |
| *Carinotetraodon lorteti* | NC015350.1 | 16456 | (Yamanoue et al. 2011) |
| *Tetractenos glaber* | NC015347.1 | 16524 | (Yamanoue et al. 2011) |
| *Lagocephalus sceleratus* | NC015340.1 | 16442 | (Yamanoue et al. 2011) |
| *Lagocephalus inermis* | PZ025842.1 | 16506 | (unpublished) |
| *Auriglobus modestus* | NC015348.1 | 16807 | (Yamanoue et al. 2011) |
| *Leiodon cutcutia* | NC015365.1 | 16624 | (Yamanoue et al. 2011) |
| *Colomesus asellus* | NC015368.1 | 16479 | (Yamanoue et al. 2011) |
| *Colomesus psittacus* | NC015370.1 | 16470 | (Yamanoue et al. 2011) |
| *Sphoeroides pachygaster* | NC010960.1 | 16444 | (Yamanoue et al. 2009) |
| *Carinotetraodon salivator* | NC015352.1 | 16437 | (Yamanoue et al. 2011) |
| *Omegophora armilla* | NC015349.1 | 16471 | (Yamanoue et al. 2011) |
| *Chelonodon pleurospilus* | NC015369.1 | 16468 | (Yamanoue et al. 2011) |

Figure S1. Sequencing depth distribution across all genomic positions of the *Tetraodon pustulatus* mitogenome. Raw Illumina NovaSeq 6000 reads were filtered and mapped to the circular assembly, with an average coverage depth of 1891×. The X-axis represents the mitogenomic position (bp), and the Y-axis represents sequencing depth (×).

**References**

Anderson, S., A. T. Bankier, B. G. Barrell, M. H. de Bruijn, A. R. Coulson, J. Drouin, I. C. Eperon, D. P. Nierlich, B. A. Roe and F. Sanger (1981). "Sequence and organization of the human mitochondrial genome." Nature **290**(5806): 457-465.

Cao, L., X. Song and E. Zhang (2020). "The first mitogenome of the Nile pufferfish Tetraodon lineatus from Lake Turkana in East Africa: new insights into the genus." Journal of oceanology and limnology **38**(2): 490-502.

Formenti, G., A. Rhie, J. Balacco, B. Haase, J. Mountcastle, O. Fedrigo, S. Brown, M. R. Capodiferro, F. O. Al-Ajli and R. Ambrosini (2021). "Complete vertebrate mitogenomes reveal widespread repeats and gene duplications." Genome biology **22**(1): 120.

Gu, J., X. Zhou, C. Song, Y. Wang, H. Jin, T. Lei and X. Qi (2025). "Codon Usage Bias in Mitochondrial Genomes Across Three Species of Siphonaria (Mollusca: Gastropoda)." Genes **16**(7): 747.

Han, X. P., L. Song, Q. Wang and X. J. Chen (2025). "The complete mitochondrial genome and phylogenetic analysis of Syncrossus berdmorei (Blyth, 1860)(Cypriniformes: Botiidae)." Mitochondrial DNA Part B **10**(5): 376-381.

Jiang, H., B. Liu, Z. Lü, L. Liu and L. Gong (2018). "The complete mitochondrial genome of Lagocephalus sceleratus (Tetraodontiformes; Tetraodontidae) and phylogenetic studies of Tetraodontidae." Mitochondrial DNA Part B **3**(2): 866-867.

Liu, Y., Q. Zhou, H. Liu, C. Li and A. Tong (2016). "The complete mitochondrial genome sequence of Takifugu flavidus (Tetraodontiformes: Tetrodontidae)." Mitochondrial DNA Part A **27**(1): 613-614.

Olaosebikan, B.D. (2020). *Tetraodon pustulatus*. The IUCN Red List of Threatened Species 2020: e.T182123A135032708.

Silva-Pinheiro, P. and M. Minczuk (2022). "The potential of mitochondrial genome engineering." Nature Reviews Genetics **23**(4): 199-214.

Wang, Z., Y. Li, Z. Zhang, L. Lin and T. Gao (2016). "The complete mitochondrial genome of the tessellated leatherjacket Thamnaconus tessellatus (Tetraodontiformes: Monacanthidae)." Mitochondrial DNA Part A **27**(1): 248-250.

Wei, Z., B. Zhao, X. Lin, C. Yin, S. Xu and X. Dai (2022). "The complete mitochondrial genome of Neolissochilus stracheyi (Osteichthyes: Cyprinidae)." Mitochondrial DNA Part B **7**(8): 1492-1493.

Yamada, A., A. Hamaguchi, H. Sakoda, M. Kakamu, H. Doi, S. Hasin and O. Arakawa (2021). "Complete mitochondrial genomes of the Southeast Asian freshwater pufferfishes, Pao abei (Roberts, 1998) and Pao suvattii (Sontirat and Soonthornsatit, 1985)(Tetraodontiformes: Tetraodontidae) and an insight into the taxonomic status of Pao species." Mitochondrial DNA Part B **6**(4): 1448-1450.

Yamanoue, Y., M. Miya, H. Doi, K. Mabuchi, H. Sakai and M. Nishida (2011). "Multiple invasions into freshwater by pufferfishes (Teleostei: Tetraodontidae): a mitogenomic perspective." PLoS One **6**(2): e17410.

Yamanoue, Y., M. Miya, K. Matsuura, S. Miyazawa, N. Tsukamoto, H. Doi, H. Takahashi, K. Mabuchi, M. Nishida and H. Sakai (2009). "Explosive speciation of Takifugu: another use of fugu as a model system for evolutionary biology." Molecular biology and evolution **26**(3): 623-629.
